# Assessment of the level of knowledge about first aid among Polish army soldiers

**DOI:** 10.3389/fpubh.2025.1716986

**Published:** 2026-01-12

**Authors:** Izabella Krenzel, Izabela Narewska

**Affiliations:** Silesian Academy, Katowice, Poland

**Keywords:** first aid, level of knowledge, Polish army soldier, public health, soldier

## Abstract

**Introduction:**

First aid is defined as the actions taken by any person to assist someone experiencing acute illness or injury. The primary goals of first aid are to save lives and prevent further injury, as well as to aid recovery. This research was conducted because knowledge of first aid is important when working alongside healthcare professionals with soldiers.

**Aim of the study:**

To examine the level of first aid knowledge among professional soldiers in the Polish Army.

**Material and methodology:**

The study was conducted with a group of 137 professional Polish Army soldiers. The study used a diagnostic survey in the form of a questionnaire. The questionnaire consisted of 29 questions, 11 of which contained socio-demographic data and information on how to improve the respondents' first aid skills. The remaining 18 questions were directly related to first aid procedures.

**Results:**

The study shows that professional soldiers' knowledge of first aid in the Polish Army is directly influenced by their level of education. Those with vocational education demonstrated significantly less knowledge than those with secondary or higher education. A second statistically significant finding was that soldiers in the officer and non-commissioned officer corps had a higher level of first aid knowledge than those in the enlisted corps.

**Conclusions:**

The study shows that Polish Army soldiers' knowledge of first aid is directly influenced by their education and position. However, having completed a cardiopulmonary resuscitation (CPR) course or having provided first aid to an injured person in the past is not important.

## 1 Introduction

The modern world is characterized by constant and dynamic evolution of biological threats. Reports of new, potentially pandemic pathogens appear almost constantly in public and scientific discourse, which perpetuates a state of global uncertainty in the area of health security. The experience of the global crisis caused by the COVID-19 pandemic has clearly demonstrated the scale of this danger and the fragility of national healthcare systems in the face of a sudden and massive number of cases.

In Poland, the SARS-CoV-2 pandemic has become an unprecedented test for crisis management mechanisms, highlighting the key role of civil-military cooperation. The Armed Forces of the Republic of Poland have been actively involved in supporting the medical sector, performing tasks ranging from logistics to directly relieving medical personnel. These experiences clearly show that in non-military crisis situations, the medical skills of soldiers take on particular importance. The ability to provide first aid efficiently and correctly is not only part of combat readiness, but also a fundamental skill that allows for effective support of the population in times of public health threats.

The above premises were the argument for the authors of this article to undertake research focusing on assessing the level of knowledge and preparation of soldiers in the field of first aid. The aim of the research was to diagnose and verify the actual competence of military personnel in this key area, which may serve as a basis for further analysis and possible optimization of military training systems in response to contemporary security challenges.

As regulated by the Act of March 11, 2022, on homeland defense, in Article 11, point “The Armed Forces may participate in combating natural disasters and eliminating their effects, anti-terrorist activities, activities related to the protection of property, search operations, rescue or protection of human health and life, protection and defense of cyberspace, clearing areas of explosive and dangerous materials of military origin and their disposal, as well as in the implementation of crisis management tasks.” It is clear from this that Polish Army soldiers can actively cooperate with other services, including medical services. Therefore, their level of knowledge about first aid is important in the context of performing tasks together with healthcare workers (1).

The aim of this study is to examine the level of knowledge about first aid among active Polish Army soldiers, taking into account various variables such as: years of service, the corps to which the soldier belongs, the type of military service in which he serves, and whether he has completed a Qualified First Aid Course, which is optional for active duty professional soldiers of the Polish Army. It is worth noting that, in accordance with the Medical Rescue Act, a soldier who wishes to obtain the title of rescuer must complete a Qualified First Aid course (2).

To become a professional soldier, you must have Polish citizenship, an unblemished reputation, and demonstrate loyalty to the Republic of Poland. A person applying to become a professional soldier must also have the appropriate qualifications and the physical and mental capacity to perform professional military service. After meeting the above requirements, applicants for professional military service must submit an application to the selected Military Recruitment Center, where they will undergo a series of physical and mental tests to determine their fitness for military service. As a result, the volunteer will receive a certificate of fitness for service and a draft card. The volunteer will then undergo 27 days of basic training, after which they will take the military oath. If, after completing all the above steps, they decide to become a professional soldier, they must proceed to the second stage of training, known as specialist training, which lasts up to 11 months (1, 3, 4).

According to the Central Statistical Office in its publication Mały Rocznik Statystyczny Polski 2024 (Small Statistical Yearbook of Poland 2024), the Armed Forces of the Republic of Poland number 180,800 personnel, of whom 134,300, or 74.3%, are professional soldiers. Among professional soldiers, there are 38,200 women and 96,100 men, which in summary gives 28.4% women and 71.6% men serving as professional soldiers in the Polish Army (5).

The professional staff of the Armed Forces is divided into three main corps:

- The officer corps, which includes:

- junior officers
- senior officers
- generals and admirals

- The non-commissioned officers corps, which includes:

- junior non-commissioned officers
- non-commissioned officers
- senior non-commissioned officers

- The enlisted personnel corps

## 2 Materials and methods

The aim of the analysis was to determine the level of first aid knowledge among respondents.

### 2.1 Research tool and procedure

The diagnostic study was conducted as an online survey between November 2024 and February 2025. The survey was conducted using an online form among a group of 137 (*N* = 137) professional soldiers of the Polish Army. The survey was posted on portals intended for soldiers, and participants were asked to complete the questionnaire voluntarily.

The survey consisted of 28 questions, including 11 questions collecting sociodemographic data (age, gender, and military rank) and information about respondents' first aid training. The remaining 17 questions aimed to assess the level of theoretical first aid knowledge among professional soldiers.

Only fully completed questionnaires were used for analysis. No questionnaires were rejected during the data cleaning stage, as all questions were marked as mandatory during data collection and online questionnaire preparation. If a participant chose not to answer or forgot to answer any question, they could not submit the questionnaire.

### 2.2 Survey design and question selection

The questions assessing theoretical knowledge were developed by a research team consisting of a nurse and a public health specialist. The list of questions (covering the topics listed below) was developed based on:

1. Practical experience: Knowledge derived directly from the research team's experience working in the civilian healthcare system during the COVID-19 pandemic. This period forced soldiers to assume a supportive role in medical facilities (hospitals, ambulance transport points, vaccination centers), where civilian emergencies were the most common.
2. Literature analysis: An analysis of literature and guidelines indicating typical knowledge gaps in Basic Life Support (BLS) knowledge among the general public was included.

The selected range of questions (compulsory first aid, assessment of basic vital signs, BLS algorithm, AED, choking, stroke, musculoskeletal injuries, and burns) intentionally focused on the universal basics of BLS. This was intended to assess soldiers' knowledge readiness for a supportive role in the civilian healthcare system, rather than their combat readiness. For this reason, advanced military procedures [e.g., Tactical Combat Casualty Care (TCCC)], such as massive hemorrhage control or mass casualty triage, were intentionally omitted, as they were not a priority for hospital support.

### 2.3 Statistical analysis

Calculations were performed using IBM SPSS Statistics version 30.0.

Theoretical knowledge was assessed by awarding respondents 1 point for each correct answer. The percentage of correct answers was then calculated for each respondent, and based on this, they were classified into knowledge levels, using a scale from 2 (unsatisfactory) to 5 (very good). The table presents individual questions and their answers, with the correct answer to each question highlighted in green.

Results are presented using absolute numbers (*n*) and percentages (%). Relationships between categorical variables (e.g., knowledge level and training level) were assessed using crosstabulations and the chi-square test (chi^∧^2). The significance level was *p* < 0.05.

The table below (Table 1) presents answers to socio-demographic questions and information on first aid qualifications.

**Table 1:** Description of the study population.

**Variable**		***n***	**%**
Gender	Woman	42	30.66
	Man	95	69.34
Age	Under 20	1	0.73
	20–30	42	30.66
	30–40	68	49.64
	40–50	24	17.52
	Over 50	2	1.46
Years of service	Under 1 year	7	5.11
	1–5 years	35	25.55
	5–10 years	41	29.93
	10–20 years	45	32.85
	Over 20 years	9	6.57
Which corps do you belong to?	Enlisted personnel	64	46.72
	Non-commissioned officers	58	42.34
	Commissioned officers	15	10.95
Your education	Vocational	11	8.03
	Secondary	71	51.83
	Higher	55	40.15
Do you have a certified first aid course?	No	32	23.36
	Yes	105	76.64
Where did you complete your certified first aid course? (question for persons who have completed a certified first aid course)	At secondary school	2	1.90
	Privately	11	10.48
	Outside military service (e.g., previous place of work)	9	8.57
	As part of military service	83	79.05
In which year did you complete a certified first aid course? (question for persons who have completed a certified first aid course)	Before 2020	57	54.29
	2020	8	7.62
	2021–2022	23	21.91
	2023–2024	17	16.19
Do you receive regular first aid training as part of your military service?	No	34	24.82
	Yes	103	75.18
As part of your military service, have you ever been in a situation where you had to administer first aid?	No	70	51.10
	Yes	67	48.91
During first aid training, classes were held	Practical	3	2.19
	Theoretical	12	8.76
	Theoretical and practical	122	89.05

## 3 Results

Below is a quantitative and percentage breakdown of the respondents' answers to the questions. These questions were designed to directly test the respondents' knowledge of first aid. For ease of reference, the correct answers to each question are highlighted in green in Table 2.

**Table 2:** Answers to questions about first aid knowledge.

**Question**	**Answers**	***n***	**%**
How long should it take to assess the breathing of an unconscious person?	5 s	8	5.84
	10 s	118	86.13
	30 s	5	3.65
	60 s	6	4.38
Please indicate the correct ratio of chest compressions to rescue breaths when performing CPR on an adult	2:30	8	5.84
	30:2	125	91.24
	30:3	1	0.73
	4:20	3	2.19
Please indicate the correct ratio of chest compressions to rescue breaths when performing CPR on an adult?	80–90	18	13.14
	130–140	13	9.49
	100–120	106	77.37
What is an AED?	Automatic internal defibrillator	7	5.11
	Automatic external defibrillator	127	92.70
	Laryngeal mask	1	0.73
	Chest compression system	2	1.46
What is the basic procedure for dealing with an unconscious person?	Check the body for injuries	5	3.65
	Check if they are breathing	83	60.58
	Check if their airways are clear	27	19.71
	Place them in the recovery position	22	16.06
What should we do in the event of choking (first action)?	Strike the area between the shoulder blades 5 times vigorously	37	27.01
	Call for medical assistance	5	3.65
	Check what is causing the choking	16	11.68
	Perform the Heimlich maneuver.	79	57.66
What does BLS stand for?	This is the algorithm for basic resuscitation procedures	92	67.15
	Type of pressure dressing	6	4.38
	Diagram to help assess the patient's condition correctly	29	21.17
	Device used to perform indirect heart massage	10	7.30
Who can use an AED?	Every person	115	83.94
	Every person who has completed a first aid course	14	10.22
	A person with medical training (doctor, nurse, paramedic)	6	4.38
	Only a paramedic in the Emergency Medical Services (EMS)	2	1.46
Can you recognize the first signs of a stroke?	No	25	18.25
	Yes	61	44.53
	Don't know	51	37.23
A stroke is not a symptom of	Increased blood glucose levels	77	56.20
	Speech disorders	37	27.01
	Vision disorders	9	6.57
	Dizziness	14	10.22
Which of the following heart rhythms qualifies for defibrillation?	Electrical activity without pulse	15	10.95
	Asystole	52	37.96
	Ventricular tachycardia without pulse	50	36.50
	Regular sinus rhythm	20	14.60
In what circumstances can we discontinue resuscitation efforts?	After 10 min from the start of resuscitation efforts, and the measures taken are ineffective	2	1.46
	After 5 min from the start of resuscitation efforts, and the measures taken are ineffective	2	1.46
	During rain	4	2.92
	Upon arrival of the emergency medical services (EMS)	129	94.16
When is Pott's rule/principle applied?	When assessing consciousness	18	13.14
	When a fracture is suspected, in order to immobilize the limb	92	67.15
	When checking the degree of burns	12	8.76
	When clearing the airways	15	10.95
In the event of muscle, bone or joint injuries, in order to reduce swelling, we can:	Pour water over the injured area.	2	1.46
	Apply a cool compress	116	84.67
	Apply a warm compress	1	0.73
	Apply an elastic bandage dressing	18	13.14
Who in Poland has the right and obligation to provide first aid to an injured person within the scope of their competence?	Active military personnel	1	0.73
	Every citizen	127	92.70
	Persons with appropriate qualifications	7	5.11
	Persons practicing a medical profession	2	1.46
Seeing the following on the victim's body: redness, blisters filled with serous fluid, and tissue swelling accompanied by severe pain. You will classify this burn as:	First-degree burn	56	40.88
	Second-degree burn	57	41.61
	Third-degree burn	20	14.60
	Fourth-degree burn	4	2.92
Please estimate the percentage of the victim's body surface area affected by burns to both upper limbs using Wallace's rule, also known as the “rule of nines”	1	1	0.73
	9	35	25.55
	18	91	66.42
	36	10	7.30
If the injured person has burns, you should not	Cool the wounds	4	2.92
	Lance the blisters	117	85.40
	Apply dressings	10	7.30
	Remove outer clothing if it can be removed easily	6	4.38

**Figure 1:**
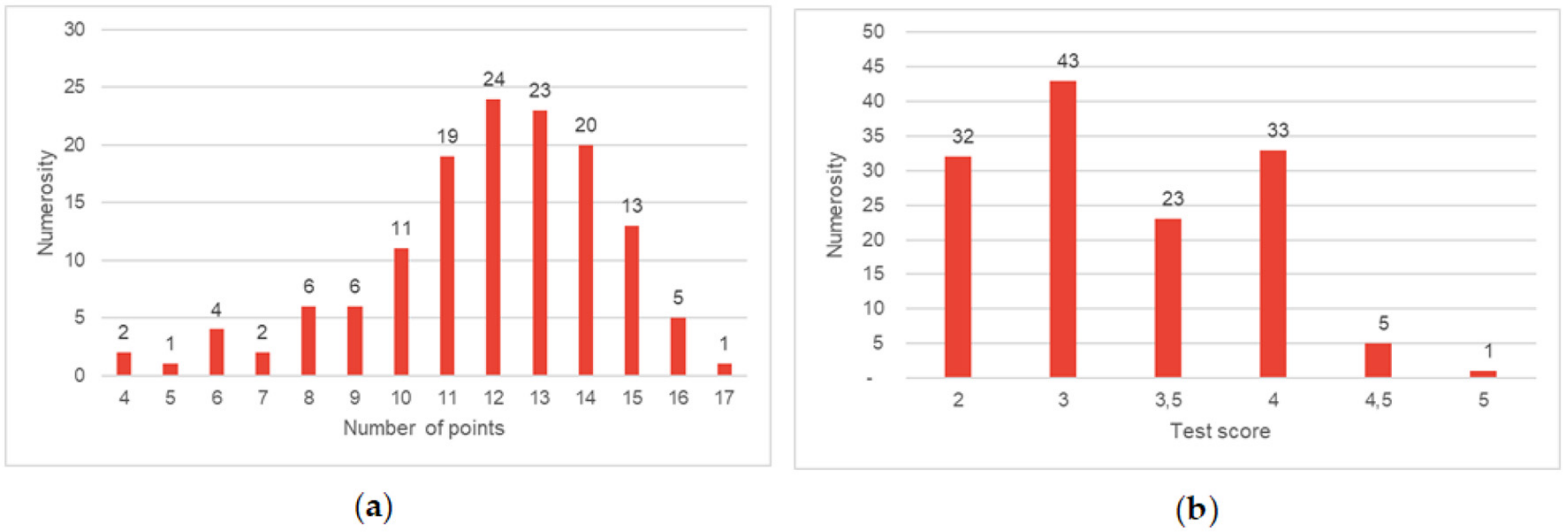
**(a)** Shows the number of respondents broken down by the number of points obtained **(b)** shows the number of respondents broken down by the score obtained in the knowledge test.

The results of the first aid knowledge test indicate (Figure 1) significant gaps in the preparation of the group of soldiers surveyed. Analysis of the score distribution (chart a) shows that although the most common score was 12 points, a significant proportion of the respondents (49 people in total) scored 11 points or less. What is more, only one person managed to score the near-maximum of 17 points, which indicates a low percentage of outstanding results.

This problem is illustrated even more clearly by graph (b), which shows the distribution of grades. The largest group (43 people) consists of soldiers who received only a satisfactory grade (3), which suggests that they have only mastered basic knowledge. Particularly alarming is the fact that as many as 32 respondents (approx. 23% of the entire group) failed the test, receiving an unsatisfactory grade (2). At the same time, only six soldiers received a grade higher than good (4.5 or 5). This distribution of results clearly shows that the level of first aid knowledge in the group surveyed is insufficient and requires urgent action to improve training programs.

The general analysis of the results presented above is a starting point for further, in-depth consideration. In order to gain a more complete understanding of the phenomenon under study, the next section of the paper presents the results of a detailed analysis, focusing on the verification of the previously formulated research hypotheses.

**Figure 2:**
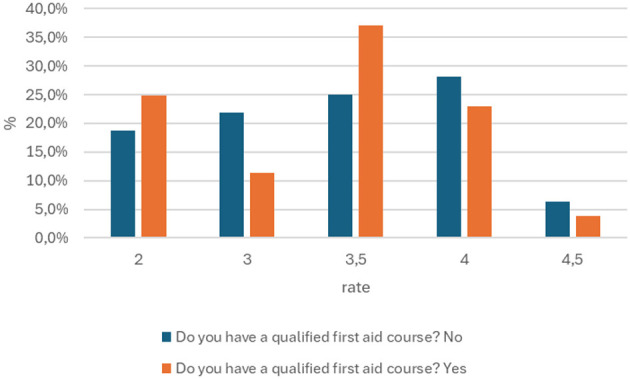
Summary of answers to the question about the course on qualified first aid with the grade obtained from the knowledge test (p = 0.409).

Based on the data provided, the hypothesis that soldiers with a qualified first aid course have the highest level of knowledge has been refuted. The value of p = 0.409 is greater than the standard significance threshold of 0.05, which means that the differences in the level of knowledge between the groups are not statistically significant (Figure 2). The analysis of the table shows that soldiers who have not completed a cardiopulmonary resuscitation (CPR) course have a higher percentage of the highest scores (4) compared to the group that has completed the course (28.1 vs. 22.9%). However, soldiers with CPR training have a significantly higher percentage of scores of 3.5 (37.1 vs. 25.0%). These results indicate some differences in the distribution of scores, but the value of p > 0.05 clearly proves that these differences are not large enough to be considered statistically significant and confirm the initial hypothesis.

It is assumed that there is a statistically significant relationship between length of service and level of knowledge in the field of first aid, including that people with longer service have a higher level of knowledge.

**Figure 3:**
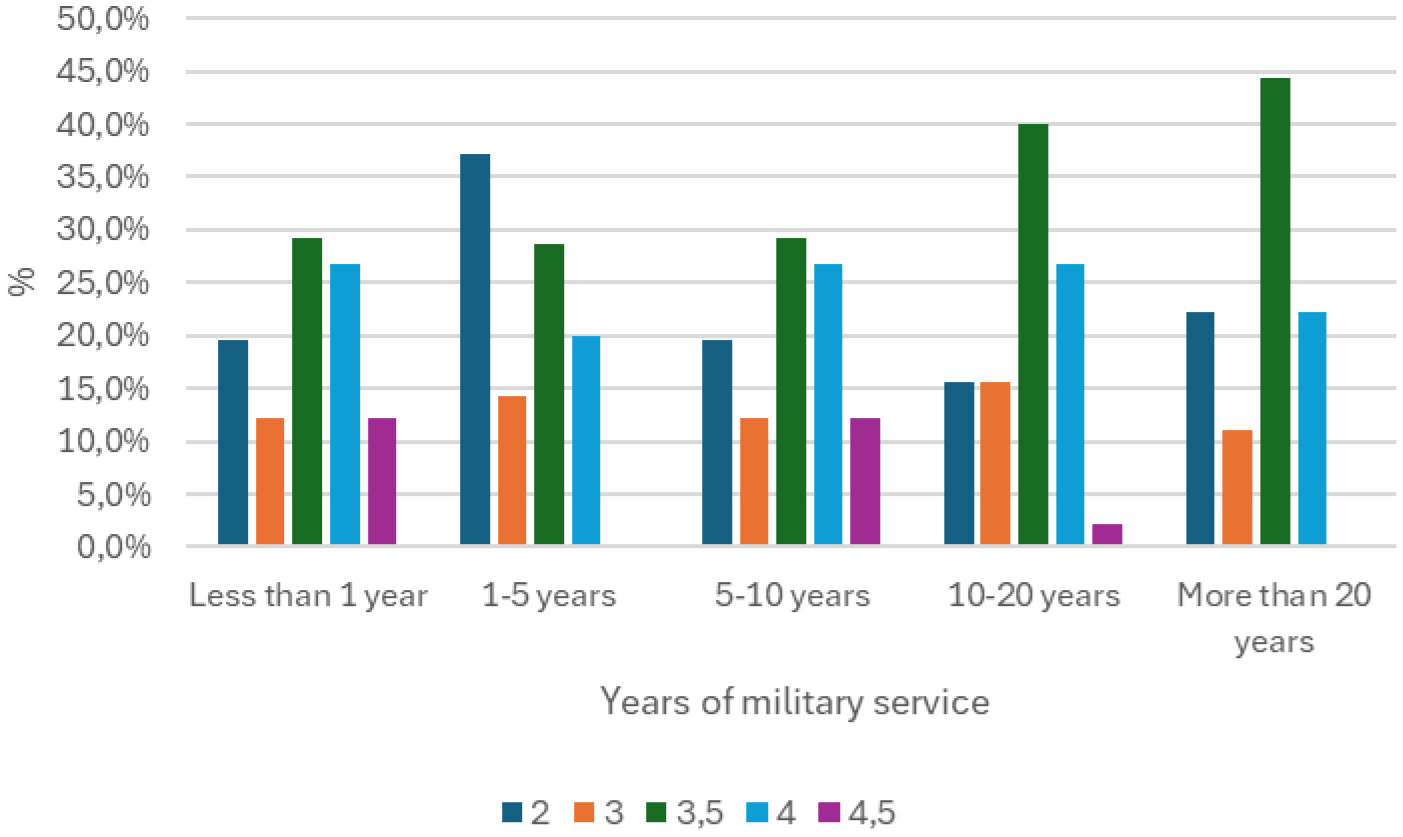
Comparison of length of service in the armed forces with the result obtained in the first aid knowledge test (p =0.503).

A statistical test was used to verify this, and the p-value obtained is 0.503. This means that there are no grounds for rejecting the null hypothesis, which states that there is no statistically significant correlation between length of service and level of knowledge. In other words, the observed differences in knowledge assessments between groups with different lengths of service are random and cannot be generalized to the entire population. In view of the above, the research hypothesis was rejected. The results of the analysis indicate that there is no statistically significant relationship between length of service and level of knowledge in the study group (Figure 3).

**Figure 4:**
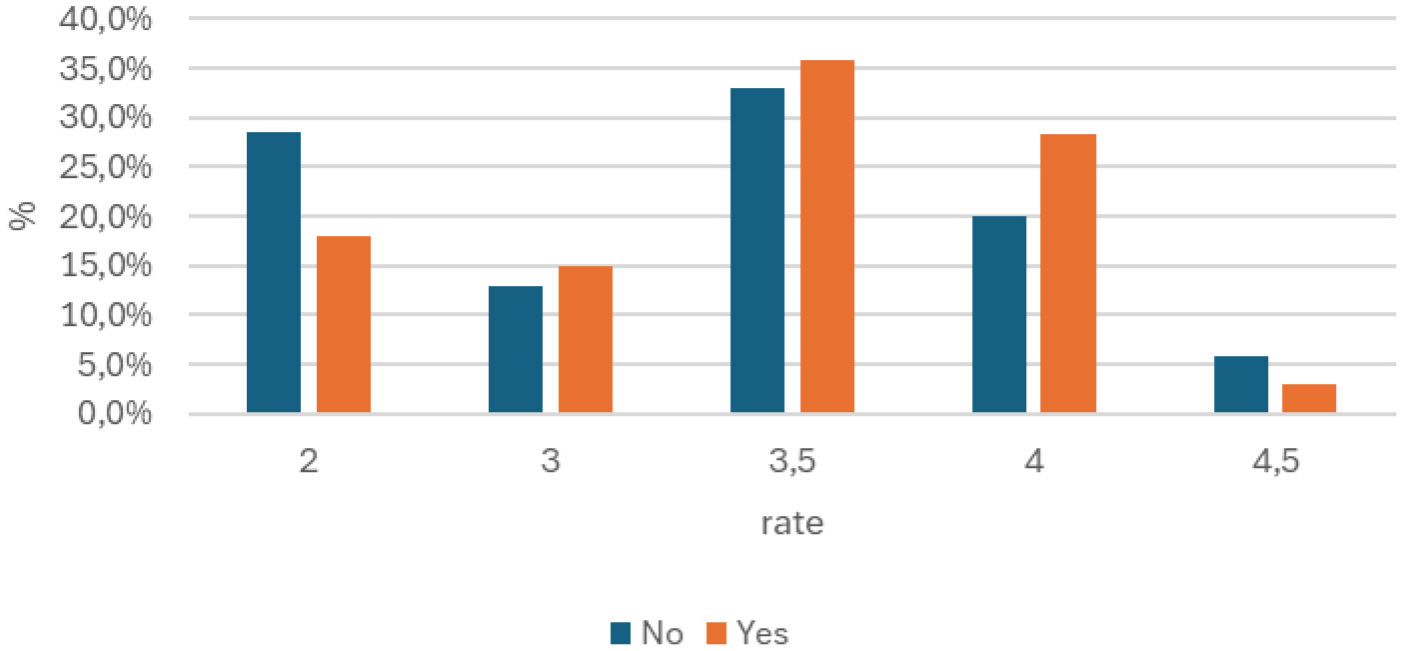
Comparison of first aid experience with the score obtained in the first aid knowledge test (p = 0.488).

The cross-tabulation table illustrates the distribution of knowledge scores (from 2 to 4.5) depending on whether the respondents had to provide first aid in the past. The total number of study participants was 137. Of this group, 67 people (approx. 49%) had such experience, and 70 (approx. 51%) did not (Figure 4). The hypothesis that people with first aid experience have greater knowledge of BLS was rejected. The chi-square test result (p = 0.488) indicates that there is no significant relationship between the fact of providing first aid and the level of knowledge in the study group. In other words, experience in a real-life situation does not translate into statistically significant higher theoretical knowledge scores.

**Figure 5:**
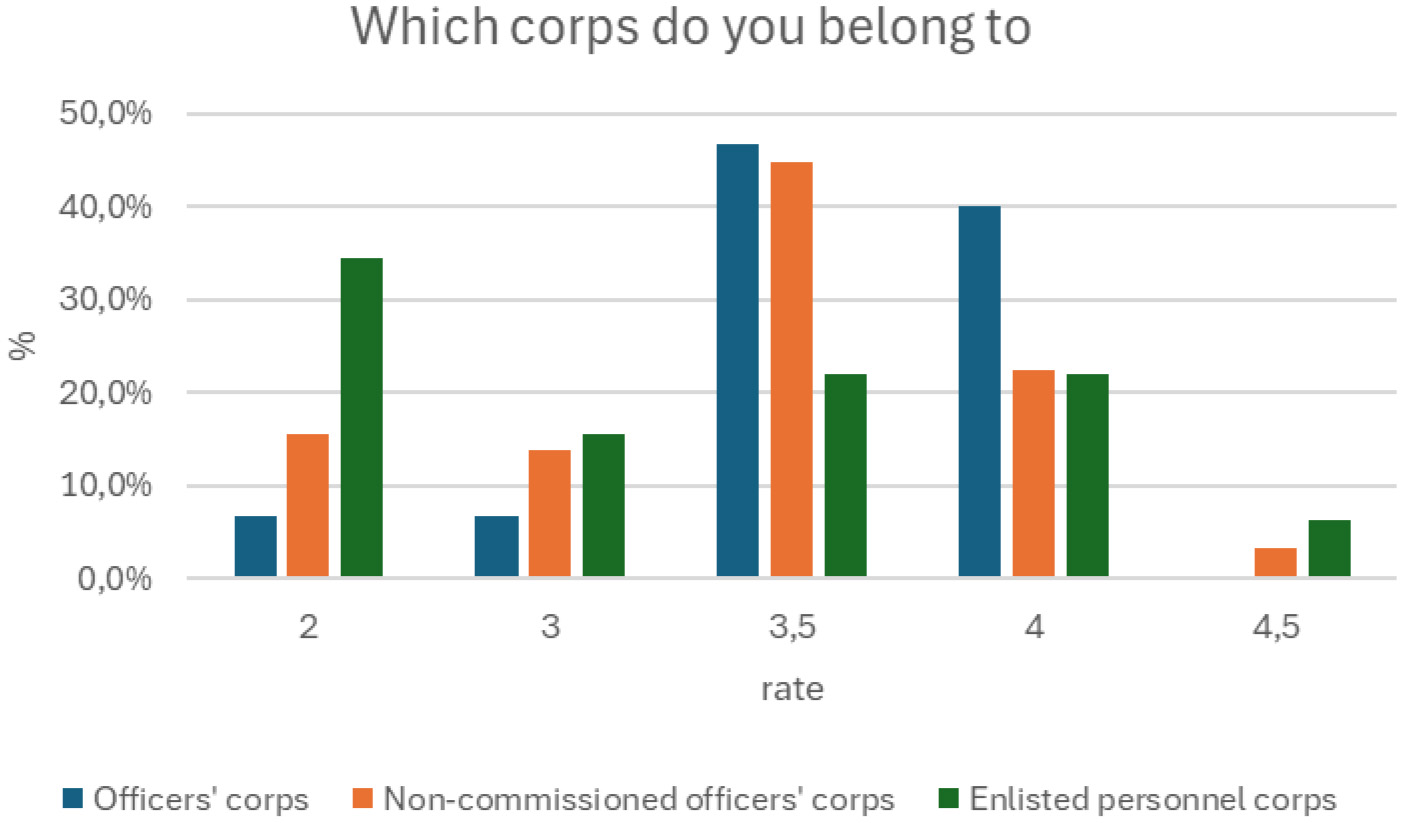
Membership in the appropriate corps based on the score obtained in the first aid knowledge test (p = 0.045).

Figure 5 shows the distribution of knowledge scores (from 2 to 4.5) among soldiers from different corps: officers, non-commissioned officers, and privates. The results are based on a survey of 137 people.

Officers corps: 40.0% of officers received a score of 4, and 46.7% received a score of 3.5. Scores of 2 and 3 were much less common (6.7% each).

Non-commissioned officers corps: 44.8% of non-commissioned officers received a score of 3.5, and 22.4% received a score of 4.

Enlisted corps: the distribution of scores is more varied. 34.4% of enlisted personnel received a score of 2, and only 21.9% received scores of 3.5 and 4.

The results of the study (p = 0.045) confirm the hypothesis that soldiers in the officer and non-commissioned officer corps have a higher level of knowledge about first aid than soldiers in the enlisted personnel corps.

**Figure 6:**
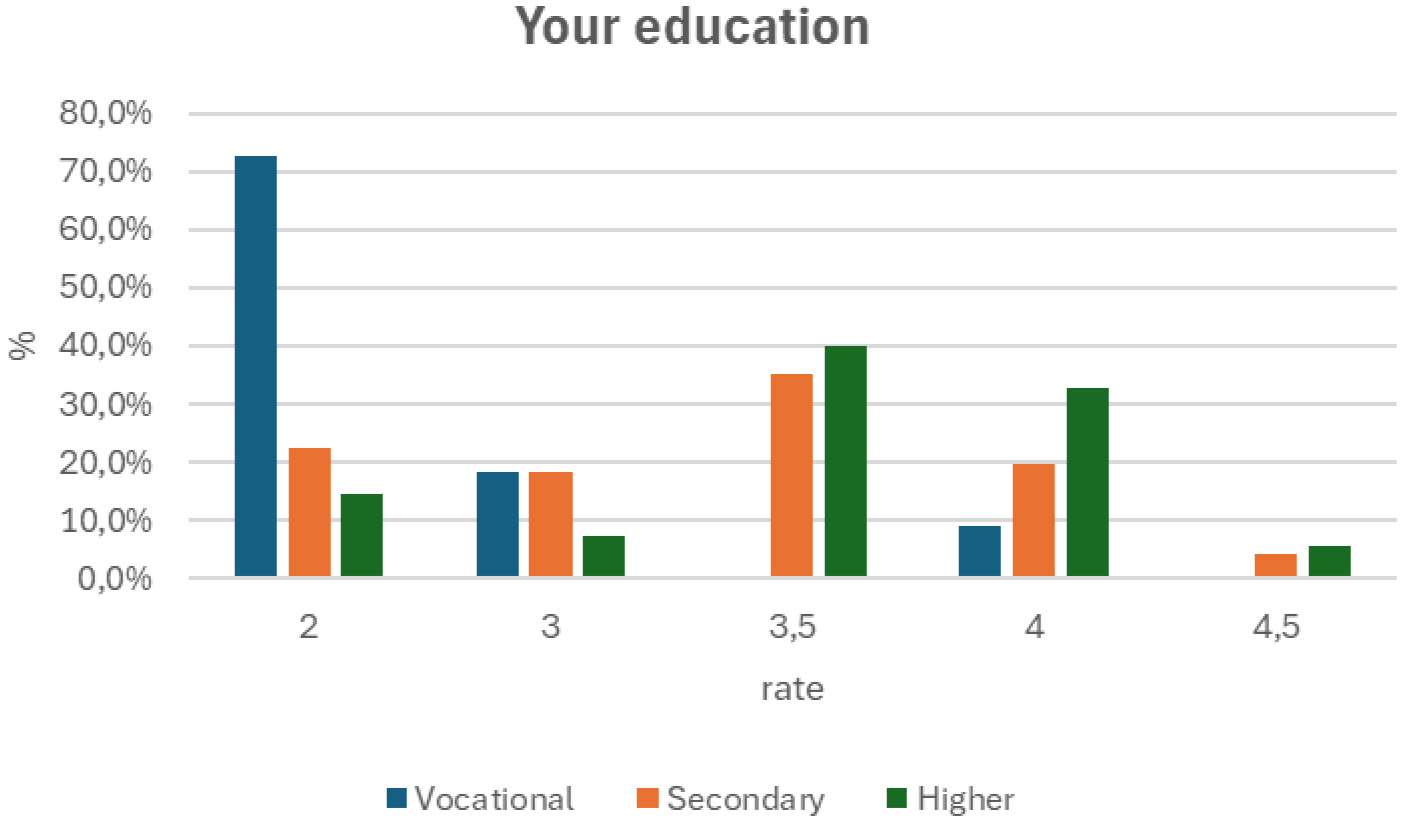
Education and first aid knowledge test score (p = 0.002).

The cross-tabulation table (Figure 6) shows the distribution of first aid knowledge scores (from 2 to 4.5) in three groups of respondents, differentiated in terms of their education: vocational, secondary, and higher. A total of 137 people took part in the study. In the vocational education group, the score of 2 predominates, obtained by 72.7% of respondents, which indicates a relatively lower level of knowledge. In the secondary education group, the most common score was 3.5 (35.2%), which suggests a moderate level of knowledge. In the group with higher education, the scores of 3.5 (40.0%) and 4 (32.7%) predominated, indicating a higher level of knowledge compared to the other groups.

The results of the analysis confirm the research hypothesis. There is a statistically significant relationship (p = 0.002) between the level of education and knowledge of first aid. In the sample studied, people with higher education obtain higher knowledge scores, which suggests that education is a factor that differentiates the level of knowledge in the field of first aid.

## 4 Discussion

The ability to provide first aid is a fundamental skill in any environment where there is an increased risk of injury. In a military context, this role takes on particular significance, going beyond the traditional understanding of tasks. Every soldier, regardless of their specialization, must be prepared to act as a “first responder,” capable of immediately providing assistance to an injured colleague or civilian. The readiness to take rescue action is crucial not only on the battlefield, where knowledge deficits can lead to preventable deaths, but also in everyday service and during non-military operations. The involvement of the Armed Forces in crisis situations, such as natural disasters, traffic accidents, or civilian support operations, requires personnel to have broad and up-to-date medical skills. According to research, psychophysical readiness, including technical and tactical skills, is an essential element of preparation for action in extreme environments.

Despite the key role that first aid skills play in safety and operational effectiveness, Polish scientific literature shows a clear deficit of comprehensive, publicly available empirical analyses that would assess the actual level of medical knowledge and skills among soldiers of the Polish Armed Forces. There is also a lack of similar studies on a global scale. The lack of systematic research covering a broad spectrum of personnel means that assessments of readiness in this area are often based on fragmentary reports. This fact confirms the existence of a research gap that hinders a holistic understanding of the scale of the problem. This points to potential systemic challenges, such as the lack of standardized evaluation tools, the confidentiality of operational data, or insufficient funding for such projects. As a result, there is a lack of objective indicators that would allow for a reliable verification of the effectiveness of current training programs and the implementation of targeted modernization measures.

Public opinion polls consistently show that the Polish Army enjoys the highest level of public trust among all state institutions. According to a CBOS survey conducted in February 2024, as many as 84% of respondents declare their trust in the army, which is an increase of 8 percentage points compared to 2022. This result significantly exceeds the trust in other public services, such as the police (72%), the government (43%), and the media (34%) (6).

One of the key factors that has contributed to the growth of public trust in the military in recent years has been its extensive involvement in the fight against the COVID-19 pandemic. As part of Operations Shield and Resilient Spring, the Polish military supported civilian medical structures on a scale unprecedented since 1989, involving up to 20,000 soldiers in these activities. Specific activities included medical transport using helicopters and planes, transport of medical equipment and ventilators, organization of temporary field hospitals, and direct support for staff and residents in social welfare homes, including taking swabs. This humanitarian face of the army, visible in rescue and relief operations in peacetime, has significantly contributed to the positive image of the military. It has built an image of an institution that is ready not only to defend the country, but also to provide direct assistance to citizens in crisis situations. However, there is a significant paradox here. The high level of public trust, built on the basis of these rescue operations, has not been preceded or verified by an in-depth analysis of the actual level of medical knowledge of soldiers, on which such assistance should be based. According to our own research, there is a statistically significant correlation between the level of education (*p* = 0.002) and membership in the corps (*p* = 0.045) and the test results. Soldiers with higher education and those belonging to the officer and non-commissioned officer corps obtained significantly higher scores compared to the group with vocational education and privates. This suggests that these factors are key to differentiating the level of knowledge in the study group. Similar results were obtained by the authors of the study Knowledge, Attitudes and Practices of Army Soldiers on Pre-hospital Trauma Care in Matara District, Sri Lanka. The study aimed to describe the level of knowledge, attitudes, and practices in pre-hospital trauma care (PHTC) among soldiers in the Matara district of Sri Lanka. A total of 266 soldiers participated in the study, and a self-administered questionnaire was used to assess their knowledge and practices, with results divided into “poor” and “good” categories with a threshold of 50%. The overall level of knowledge was poor, affecting 78.6% of the respondents. Knowledge of the “golden hour” in trauma, triage systems, and cardiopulmonary resuscitation (CPR) was particularly low. Older age, higher income, and higher education levels were found to be statistically significantly associated with better knowledge. No significant relationship between knowledge and length of service (7).

Insufficient first aid knowledge in the military poses a direct threat to operational capabilities and personnel safety. According to Tactical Combat Casualty Care (TCCC) guidelines, massive hemorrhage is the most common cause of preventable deaths on the battlefield. Rapid and correct intervention to stop bleeding is therefore crucial to saving lives. Deficiencies in this area, combined with inadequate self-assessment, can have tragic consequences, leading to deaths both in combat and during civilian support operations (8, 9).

During the 27-day basic training, soldiers learn “how to provide first aid training in the Polish Army takes place at several levels. Already first aid on the battlefield as part of self-help and peer assistance.” Specialized medical training is provided by institutions such as the Military Medical Training Center (WCKMed) in Łódź and the Military Academy of Land Forces (AWL). WCKMed offers intensive courses, including 66 h of Qualified First Aid (KPP) training, which ends with a theoretical exam requiring a minimum of 90% correct answers and a practical exam. WCKMed also runs Combat Lifesaver (CLS) courses, which require recertification every 12 months (10–12). Although there are rigorous and formalized training programs for selected groups of soldiers, there is a lack of clarity regarding the systematic and widespread implementation of these standards throughout the army. High exam requirements, such as a 90% pass rate, seem to be at odds with the generally “insufficient” level of knowledge identified in the reports analyzed. This may suggest that the problem lies not in the training standards themselves, but in their distribution, accessibility, or effectiveness in maintaining knowledge in the long term. Recertification, although formally required, may not be universally enforced, leading to a rapid decline in acquired skills (13).

Unlike the Polish model, many NATO armies base their medical training systems on standardized and hierarchical Tactical Combat Casualty Care (TCCC) protocols. The TCCC approach, based on medical algorithms adapted to combat conditions (e.g., MARCH, replacing C-A-B-C), is widely used (8, 14).

Analysis of foreign systems:

United States: The US Army uses a three-level training system. The first level is “buddy aid”—basic first aid that every soldier learns. The second is a 40-h Combat Lifesaver (CLS) course for non-medical personnel, designed to bridge the gap between basic aid and professional medical rescue. The third level is a 16-week Combat Medic course for professional rescuers (8).

United Kingdom: The British Army has introduced a new, advanced Combat Life Saver role to replace the existing Team Medica. The new role allows non-medical soldiers to perform procedures previously reserved for medics, including decompression of pneumothorax. Training in this area lasts 5 days and aims to standardize rescue procedures in accordance with NATO standards (14).

Compared to these systems, the Polish model appears to be less stratified and universal. Although CLS courses exist in the Polish Army, there is no evidence that completing them is a formal, mandatory requirement for every soldier, which is standard practice in the armed forces of the US and the UK.

A study conducted on a group of combat medics revealed an alarmingly low level of mastery of key life-saving skills. Empirical data show that performance in basic TCCC procedures is well-below expectations. In a six-question quiz testing knowledge of the current guidelines of the Committee on Tactical Combat Casualty Care (CoTCCC), medics gave an average of only 2.2 correct answers, indicating serious gaps in theoretical knowledge. Even more disturbing are the results regarding practical skills. The success rate for correctly applying a tourniquet was only 44.4%, while for the procedure of packing a wound with a hemostatic dressing, the rate dropped to 22.2%. These rates apply to medical personnel, who are the first and often crucial link in the chain of survival for wounded soldiers. The ability to perform these tasks is fundamental to preventing avoidable deaths from massive bleeding. Low effectiveness in such basic tasks signals a critical problem with the quality of training or maintenance of skills over time (15).

In contrast to the low scores in practical tests, another study, based on a survey of Army medics, showed a very high level of declared confidence. As many as 74.8% (*n* = 190) of respondents said they felt “very” or “fully” confident and prepared to provide medical care in the field. However, the same survey revealed that 25.2% (*n* = 64) of respondents did not feel so confident, and the majority of this group (84.4%) expressed a desire and need for additional training before a mission. The analysis also revealed that respondents with more mission experience or more pre-mission training felt more confident and prepared, suggesting that the quantity and quality of training influences subjective feelings of readiness (16).

In summary, analysis of available sources reveals a significant disparity: on the one hand, public trust in the Polish Army is growing, while on the other, there is a lack of reliable, systematic research assessing the actual level of soldiers' first aid readiness. This paradox points to a serious research gap that hinders informed decisions about training modernization.

Although individual studies, both Polish and foreign, suggest an insufficient level of knowledge and skills, especially in such key procedures as stopping bleeding, there is a lack of comprehensive data on the entire Armed Forces. This situation requires further in-depth empirical research. Only systematic and widespread evaluations will allow for an objective assessment of the effectiveness of current training programs and the implementation of solutions that are already standard in many NATO armies, including standardized TCCC protocols.

In light of the threats of the modern battlefield and the growing role of the military in non-military operations, investment in education and regular recertification of medical skills is crucial. Filling the research gap and implementing systemic solutions is essential so that every soldier can act as a “first responder” with full awareness and competence, saving the lives of their comrades and civilians.

## 5 Conclusions

The overall level of knowledge is insufficient. Although the most common score on the knowledge test was 12 points (out of 17), a significant proportion of respondents (over 35%) scored 11 points or less. What is more, as many as 23% of soldiers failed the test, which clearly indicates serious gaps in their preparation. Only a few (approx. 4.4%) scored above good (4.5 or 5).

No correlation between experience/service and level of knowledge. Statistical analysis showed that having a qualified first aid course (*p* = 0.409), longer service (*p* = 0.503), and personal experience in providing first aid (*p* = 0.488) has no statistically significant impact on the level of theoretical knowledge. This means that the observed differences in results between these groups are random and cannot be generalized to the entire population.

Education and Service Corps Correlate with Knowledge Level. There is a statistically significant relationship between the level of education (*p* = 0.002) and membership in the corps (*p* = 0.045) and test results. Soldiers with higher education and those belonging to the officer and non-commissioned officer corps obtained significantly higher scores compared to the group with vocational education and privates. This suggests that these factors are key to differentiating the level of knowledge in the study group.

## Data Availability

The raw data supporting the conclusions of this article will be made available by the authors, without undue reservation.
